# Supplementary material for: Rapid SABRE Catalyst Scavenging Using Functionalized Silicas
Source: Molecules. 2022 Jan 6;27(2):332. doi: 10.3390/molecules27020332 (PMC8778821; doi:10.3390/molecules27020332)
Supplement: Supplementary file 1 [file molecules-27-00332-s001.zip › molecules-1471092-supplementary.pdf]

Rapid SABRE catalyst deactivation using functionalized silicas

Thomas B. R. Robertson [1,2]*, Leon J. Clarke [2] and Ryan E. Mewis [2]

[1] Department of Natural Sciences, Faculty of Science and Engineering, Manchester Metropolitan University, Chester Street, Manchester, M1 5GD, UK; l.clarke@mmu.ac.uk (L.J.C.); r.mewis@mmu.ac.uk (R.E.M.)
[2] Current Address: School of Chemistry, University of Southampton, SO17 1BJ, Southampton, UK; t.b.r.robertson@soton.ac.uk (T.B.R.R.)
* Correspondence: t.b.r.robertson@soton.ac.uk

Contents:

S1. Characterisation of iridium species formed in the presence of ethylenediamine and diethylenetriamine — 2
S2. Optimisation of ICP-OES methodology — 6
S3. Preparation of ICP-OES samples — 9
S4. Effect of long scavenger exposure times on solution Ir content — 10
S5. Hyperpolarisation of 2-picoline with a co-ligand — 11
S6. Equation for extrapolation of scavenger quantities required — 12

S1. Characterisation of iridium species formed in the presence of ethylenediamine and diethylenetriamine

Species formed upon addition of 2 equivalents ethylenediamine to [Ir(IMes)(pyridine)$_3$(H)$_2$]

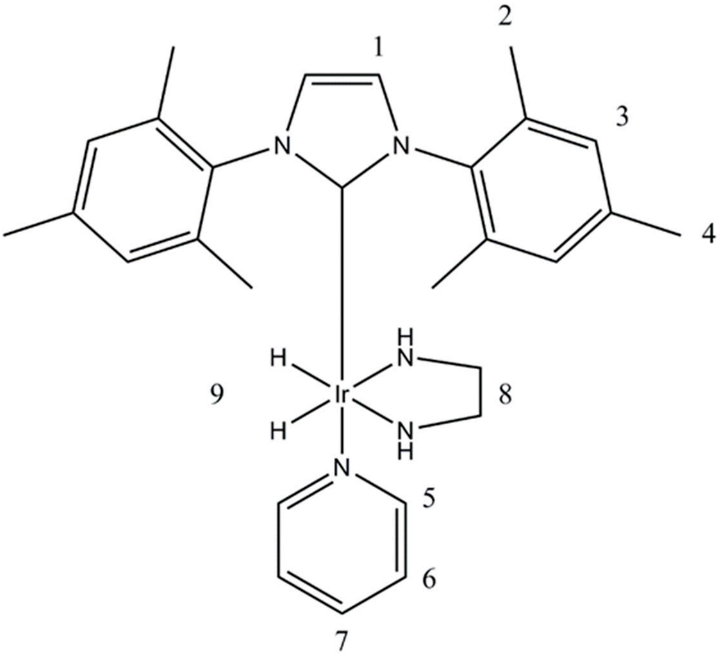

Figure S1. Major species in solution following addition of two equivalents ethylenediamine to [Ir(IMes)(pyridine)$_3$(H)$_2$].

Table S1. ^{1}H NMR characterisation data for complex shown in Figure S1.

Site number	^{1}H chemical shift/ppm
1	7.10
2	2.20
3	7.13
4	2.39
5	8.49
6	7.16
7	7.73
8	2.05
9	-21.84

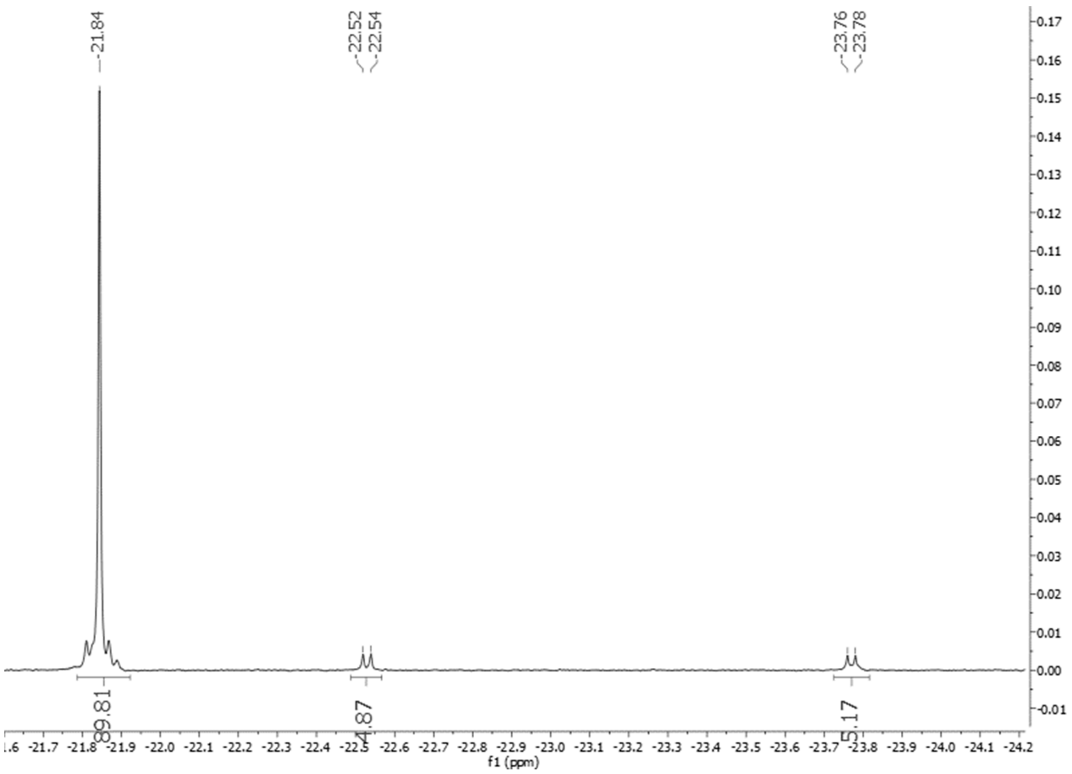

Figure S2. Hydride region demonstrating the major and minor species in solution following addition of two equivalents ethylenediamine to [Ir(IMes)(pyridine)$_3$(H)$_2$].

Species formed upon addition of 2 equivalents diethylenetriamine to [Ir(IMes)(pyridine)$_3$(H)$_2$]

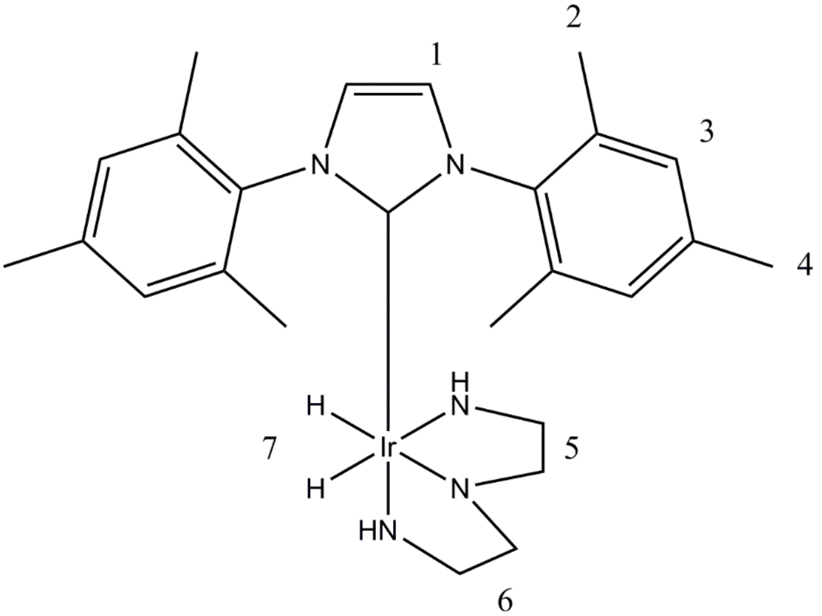

Figure S3. Majority species in solution following addition of two equivalents diethylenetriamine to [Ir(IMes)(pyridine)$_3$(H)$_2$].

Table S2. NMR characterisation data for complex shown in Figure S3.

Site number	^{1}H chemical shift/ppm
1	7.03
2	2.11
3	7.07
4	2.36
5	2.16
6	
7	-22.68

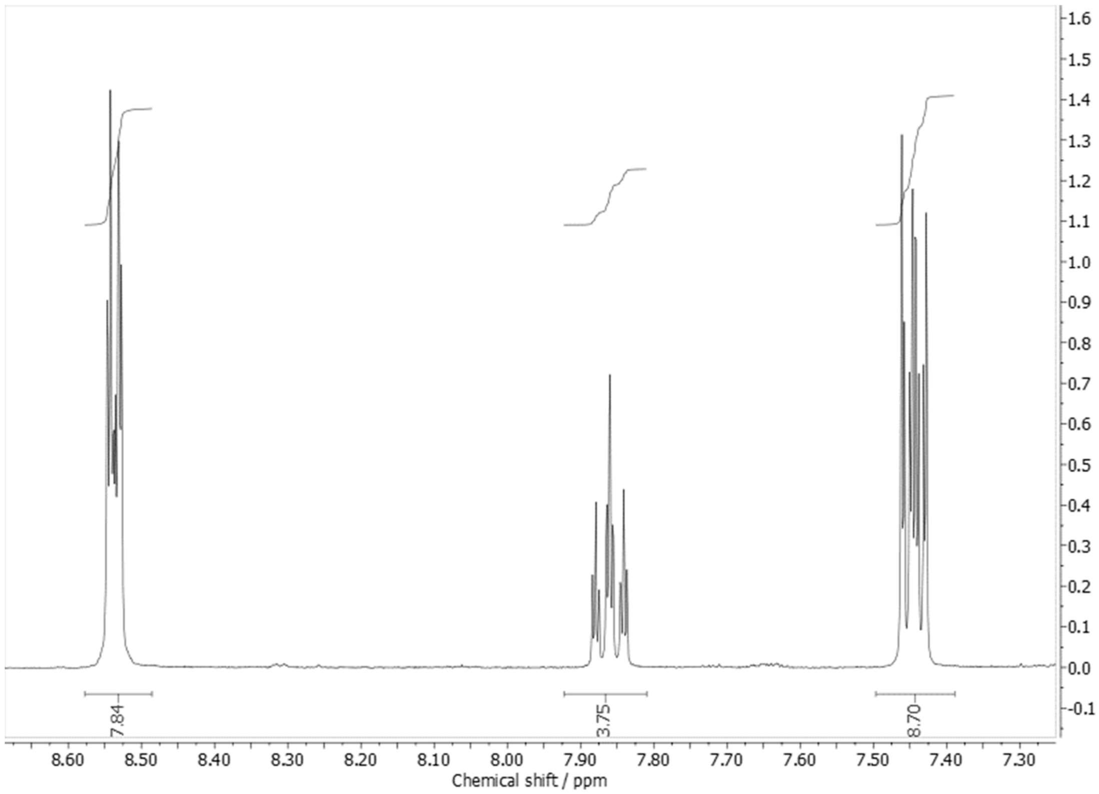

Figure S4. Aromatic region demonstrating the lack of bound pyridine peaks following addition of 3 equivalents diethylenetriamine to [Ir(IMes)(pyridine)$_3$(H)$_2$].

S2 Optimisation of ICP-OES methodology

SABRE samples contain a significant quantity of organic residue due to the catalyst-bound carbene, methanol and pyridine used in this study. Although it is not possible to matrix match the iridium-bound organics, it was possible to account for the methanol solvent and pyridine substrate used to activate the polarisation pre-catalyst **1**. To do this, a stock solution containing the quantities of pyridine and methanol representative of a SABRE sample, with 4 equivalents of pyridine used relative to **1**, was prepared. This organic-containing stock then was used to spike a range of iridium standards from 10 ppb to 10 ppm with 0-1% v/v organic spiking. These standards were then analysed in both axial and radial viewing modes to assess which ICP-OES measurement method would be most suitable for this study (figures S5 and figure S6, respectively).

These tests demonstrated that the presence of organics had a minor effect on the signal intensity observed and resultant calibration lines. Changing the ICP radiofrequency (RF) power within the working range between 1050 W and 1150 W demonstrated little effect; therefore, the ICP-OES instrument optimised value of 1175 W was used. The axial viewing mode was also utilised for further work as this provided the highest intensity and thus minimised the effect of the organic sample spiking. The standards spiked with 1% organic stock were utilised as external calibration standards for the quantitative determination of iridium concentration for this study as this was most representative of the samples measured.

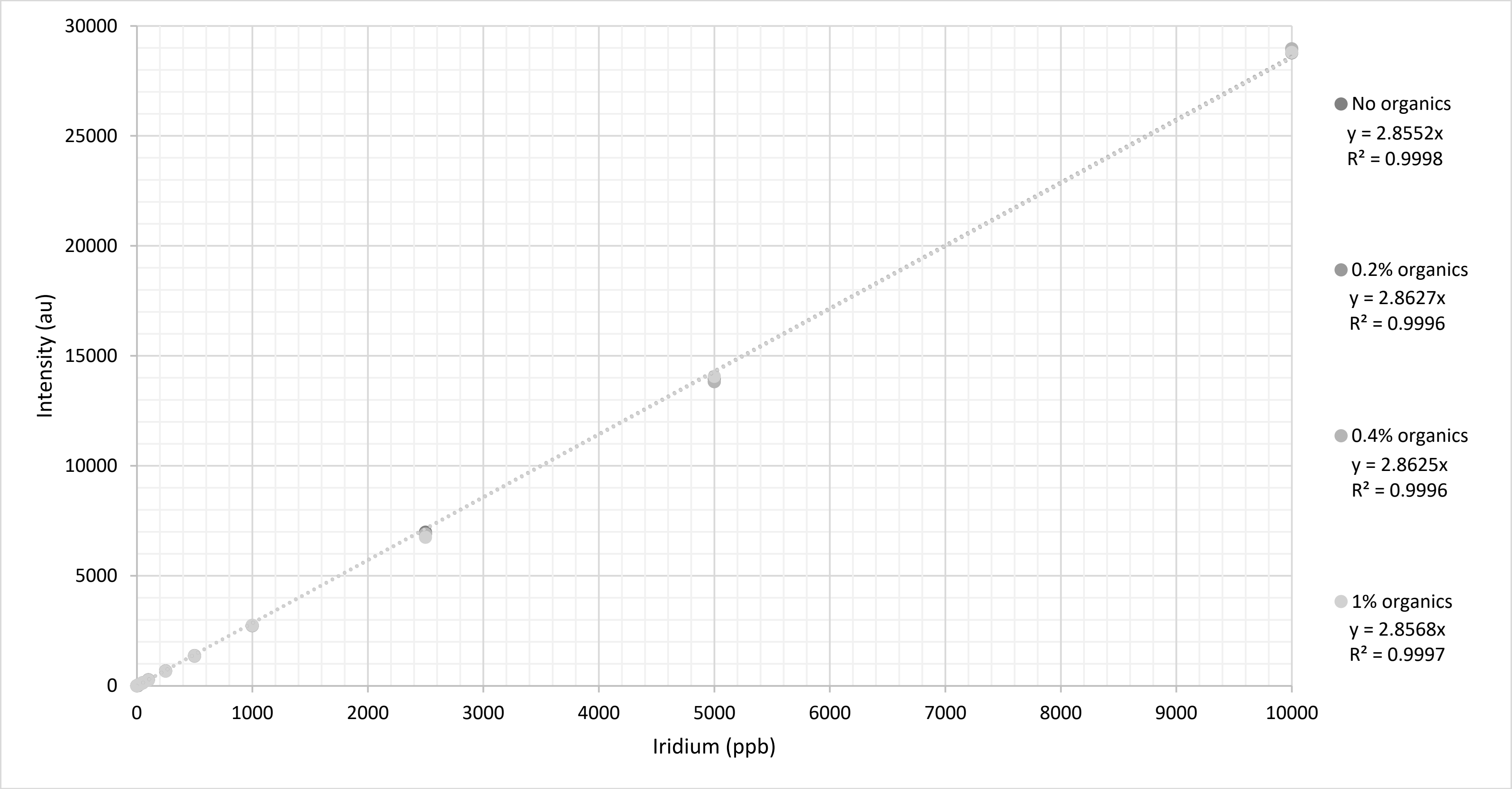

Figure S5. A calibration plot of 0 to 10 ppm iridium making use of the axial viewing mode. The stock solutions had been spiked with between 0 and 1% of an organic stock representative of the organics present in a SABRE sample.

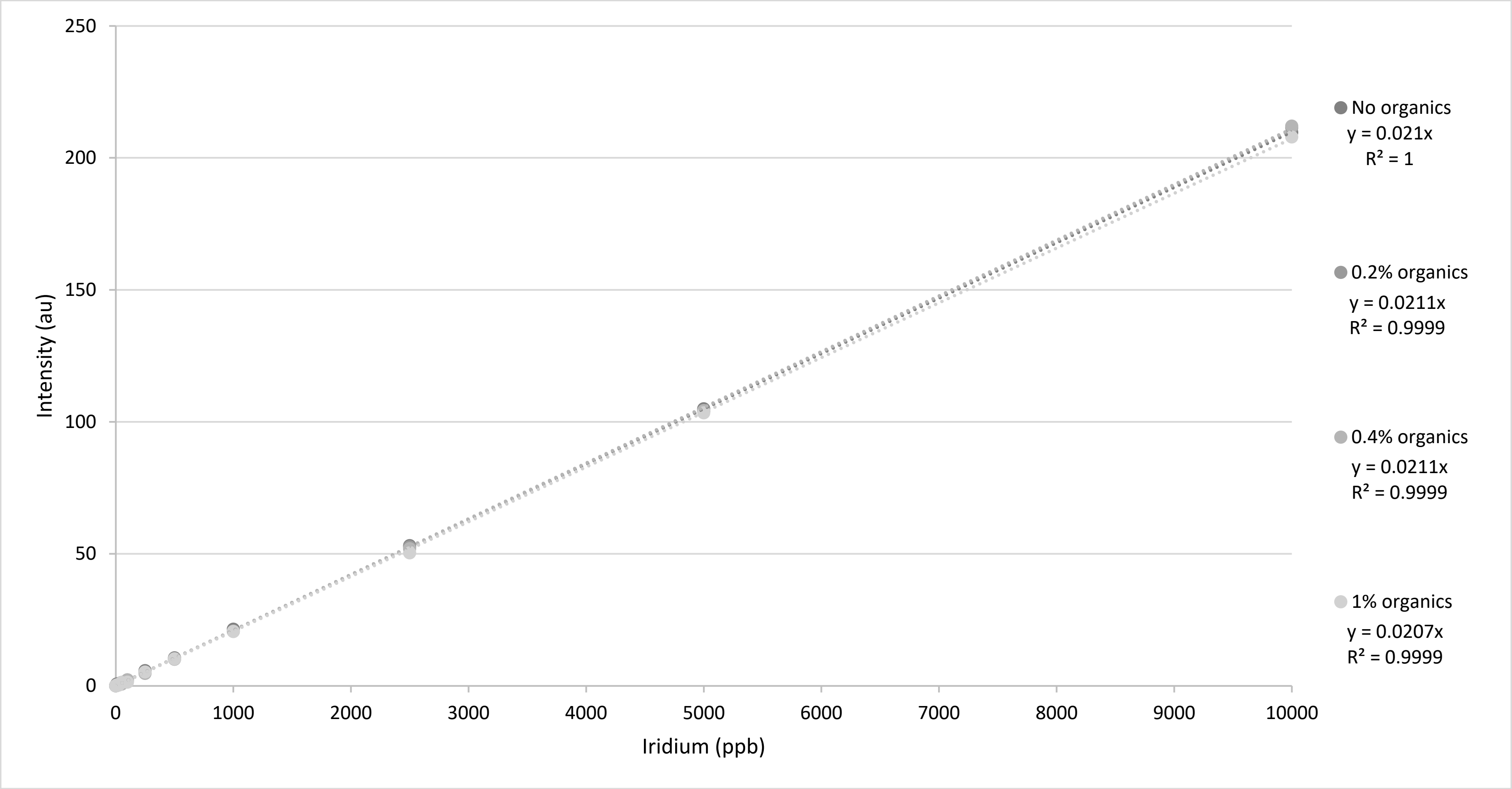

Figure S6. A calibration plot of 0 to 10 ppm iridium making use of the radial viewing mode. The stock solutions had been spiked with between 0 and 1% of an organic stock representative of the organics present in a SABRE sample.

S3 Preparation of ICP-OES samples

Scavenger particles could potentially interfere with measurement *via* blockages in the sample injection system. Therefore, it was experimentally challenging to measure the iridium remaining after very short time periods as samples required centrifuging before analysis. The time point labelled t = 0 is therefore the fastest experimentally feasible testing time, with other time points labelled for the total 'wait' time without necessary centrifuge time considered. Further details of this method are included in section **Error! Reference source not found.**, with a flow chart demonstrating the preparation of samples t = 2 mins and t = 6 mins shown in figure S7 and the actual exposure times of each sample summarised in table S3.

Table S3. Actual exposure times due to experimental limitations of ICP-OES samples. An example of the preparation steps utilised for samples t = 2 mins and t = 6 mins is detailed in figure S7.

Label	Actual exposure time
2 mins	2 mins 10 seconds
6 mins	6 mins 20 seconds
11 mins	11 mins 30 seconds
18 mins	18 mins 40 seconds
25 mins	25 mins 50 seconds
32 mins	33 mins

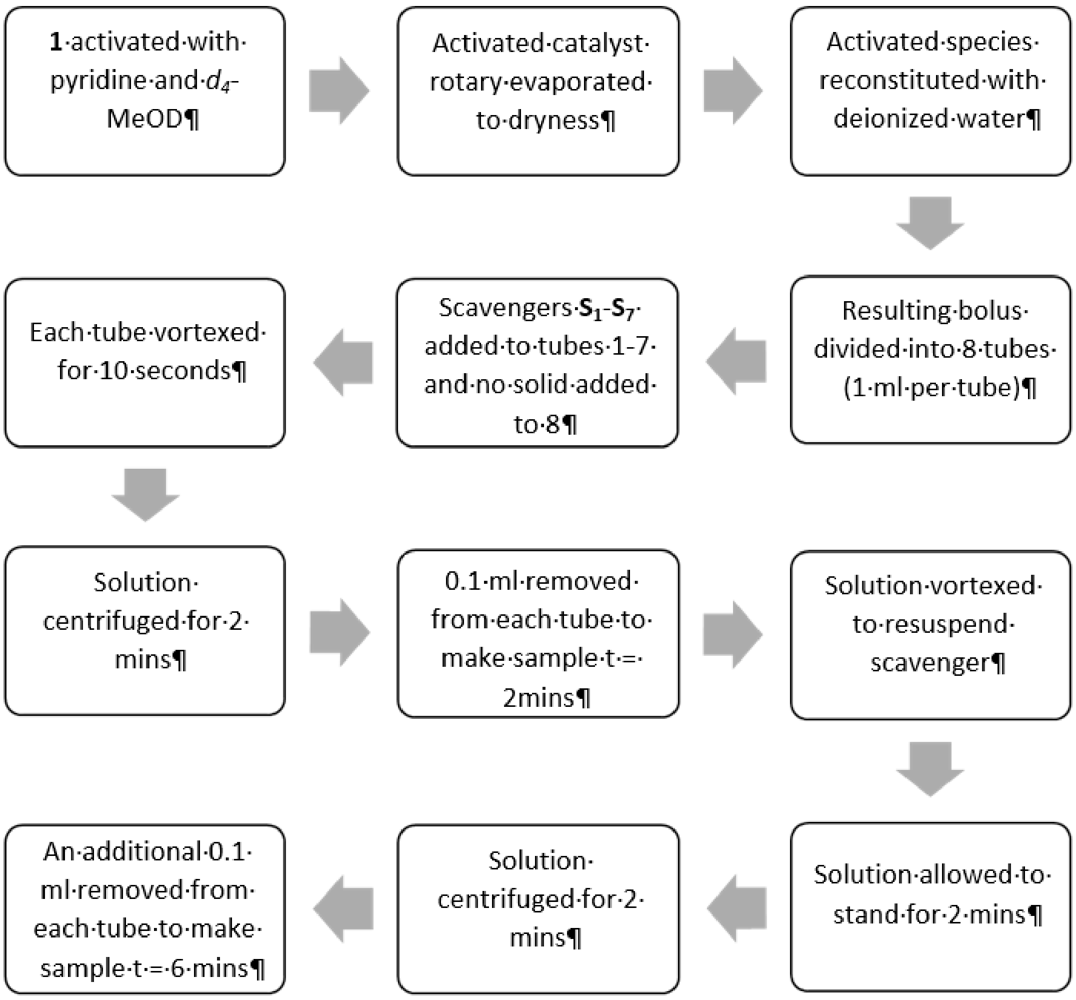

Figure S7. Flow chart demonstrating the preparation steps for ICP-OES samples t =2 mins and t = 6 mins. Additional time samples were gathered through the repetition of the vortex, wait, centrifuge, sample steps. This resulted in the actual exposure times shown in table S3.

S4 Effect of long scavenger exposure times on solution Ir content

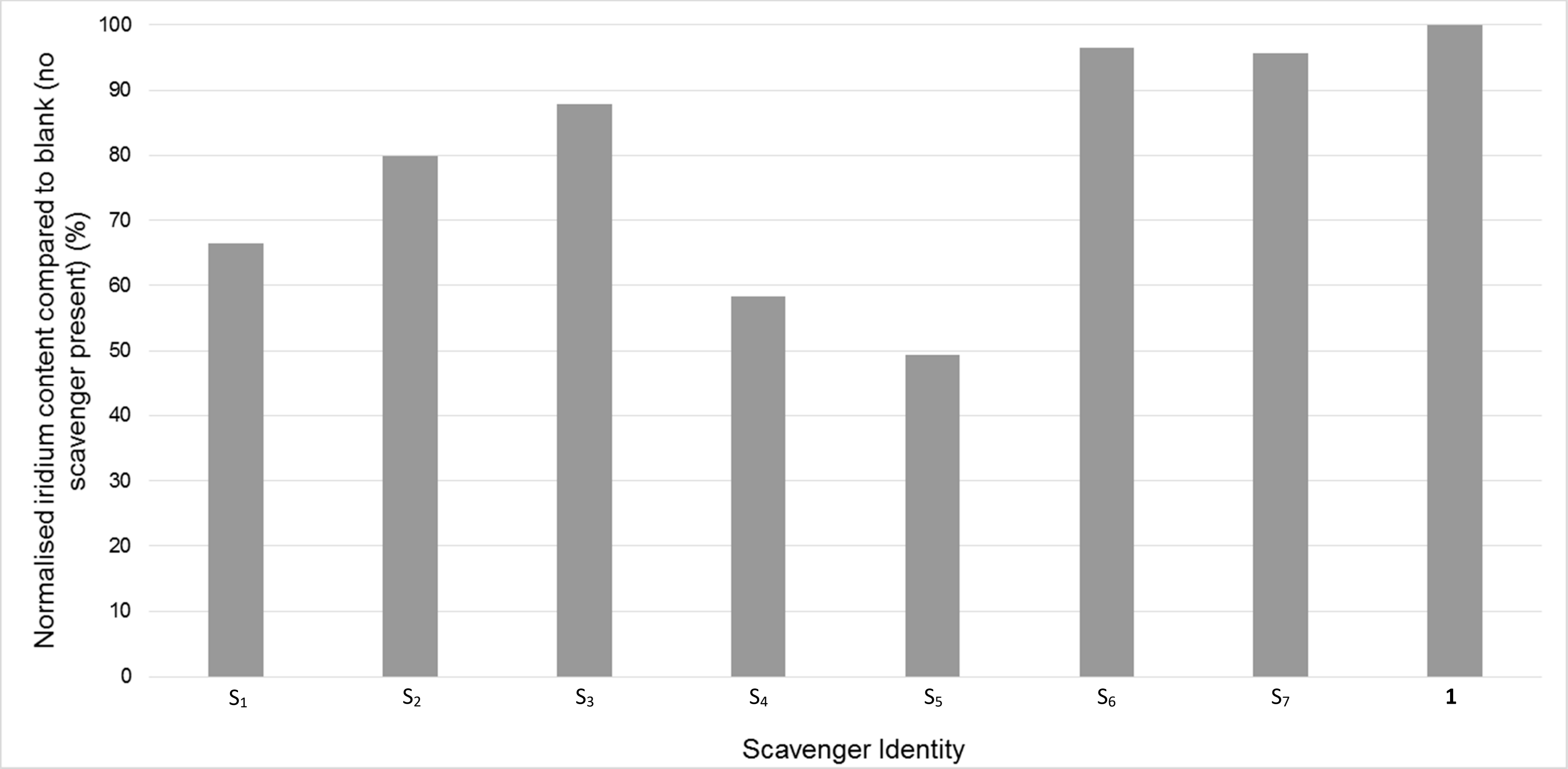

Figure S8. Iridium present in samples following the addition of 10 mg of scavengers **S_1-S_7** and with no scavenger added (**1**) after 12 days normalised to iridium content of the blank sample with no scavenger added.

S5 Hyperpolarisation of 2-picoline with a co-ligand

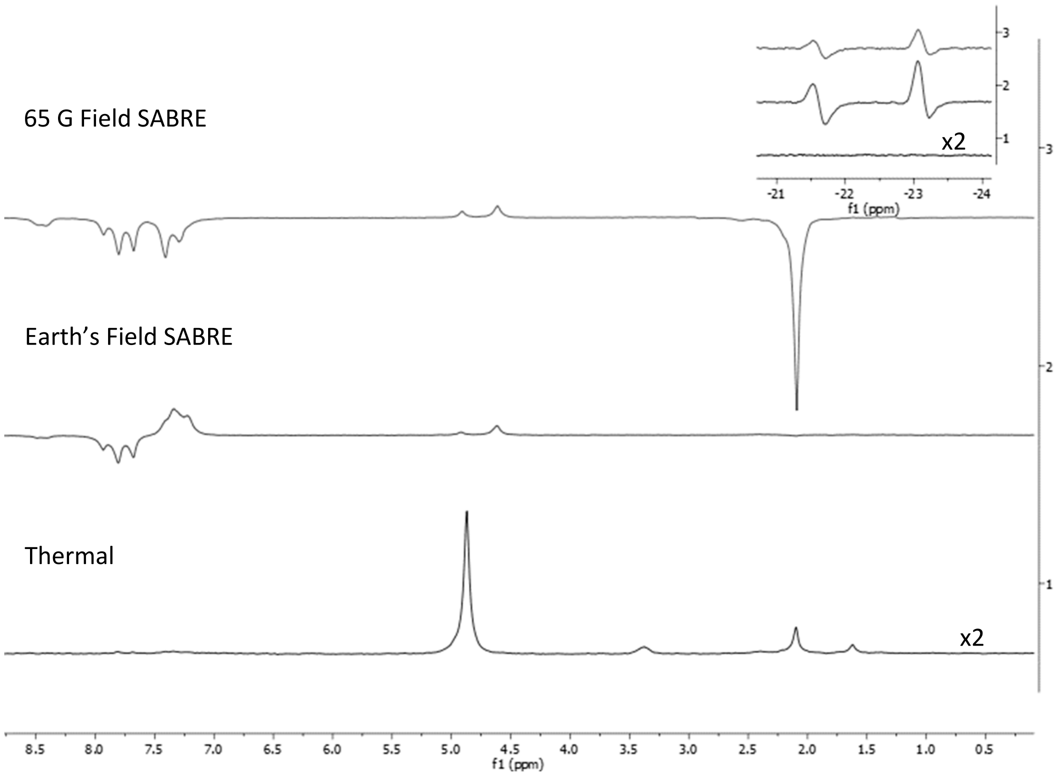

Figure S9. Spectra of 2-picoline (4 equivalents), acetonitrile (10 equivalents) relative to a single equivalent **4**. Hydride region shown as expansion. Thermal spectrum is scaled by 2-fold relative to the other spectra.

Table S4. 2-Picoline SABRE enhancements at earth's field and 65 G when acetonitrile is utilised as a co-ligand.

Magnetic field in which hyperpolarisation conducted	Proton Environment Enhancement		
	Ortho	*Meta*	*Para*
Earth's field	9.28	28.14	39.35
65 G	19.46	32.53	54.62

Utilising acetonitrile as a co-ligand in order to hyperpolarize 2-picoline resulted in SABRE being observed. This result shows relatively good enhancements and a pair of hydrides occurring at -21.69 and -23.14 ppm. The first resonance is broadly in agreement with a resonance of -20.56 which has been reported for a hydride *trans* to acetonitrile in the complex [Ir(IMes)(acetonitrile)(pyridine)$_2$(H)$_2$] suggesting this is the acetonitrile adduct within the 2-picoline containing complex.[1] The hydride *trans* to pyridine in the report by Mewis *et al.* is reported at -22.12 ppm and hydrides *trans* to ammonia have been reported at -23.61 ppm,[2] therefore it seems likely the hydride herein present at -23.14 ppm can be attributed to the hydride *trans* to 2-picoline. The polarisation reported here demonstrates that when a co-ligand is present which can pre-activate the metal complex then the functionality present in **S$_5$** is capable of binding to the iridium catalyst. In this instance pyridine pre-activated the complex as this was utilised in the preparation of the ICP-OES bolus due to the need for water solubility.[3]

S6 Equation for extrapolation of scavenger quantities required

Iridium remaining in the sample was calculated via extrapolation of figure 5 using the formula below:

$$y = me^{-nx}$$

$$\frac{Ln\left(\frac{y}{m}\right)}{-n} = x$$

Equation S1 Equation utilised for the extrapolation of scavenger quantity needed to remove enough iridium to match the value measured for deionized water *x*.

The average iridium concentration detected in n=5 deionized water blanks run before the experiments was 0.2126 ppb.

For all time points y = 1708.7e^{-nx} therefore the value of $Ln\left(\frac{y}{m}\right)$ does not change from -8.99194.
